# Associations between the perceived educational environment and burnout symptoms across multilingual medical programs: the role of academic and social self-perception

**DOI:** 10.1186/s12909-026-08786-8

**Published:** 2026-02-10

**Authors:** István Szabó, Árpád Csathó, Béla Birkás

**Affiliations:** 1 Department of Behavioral Sciences, Medical School, University of Pécs, Szigeti str. 12., Pécs, Baranya H 7624, Pécs, Hungary; 2 Quality Assurance Office, Rector’s Office, Pázmány Péter Catholic University, Budapest, Hungary; 3 Szentágothai Research Centre, University of Pécs, Pécs, Hungary

**Keywords:** Burnout, Educational environment, Self-reflective mechanism, DREEM, Medical students, International students

## Abstract

**Background:**

Burnout among medical students is a widespread problem that threatens academic performance, well-being, and professional identity. The educational environment is crucial for shaping students’ psychological resilience regarding burnout. The current study investigates how perceptions of the educational environment and reflective self-perceptions are associated with burnout symptoms among students enrolled in three different language medical programs.

**Methods:**

A cross-sectional, self-report study was conducted among 731 medical students across three language programs at a Hungarian university. Participants completed the Maslach Burnout Inventory–Student Survey (MBI-SS) and the Dundee Ready Education Environment Measure (DREEM). Language program, age, sex and study semester were also included as variables into the model and statistical analyses to check for potential meaningful differences.

**Results:**

Significant differences in burnout dimensions were observed across language groups, with international students reporting higher emotional exhaustion and lower academic efficacy. All five DREEM subscales showed moderate correlations with burnout dimensions. Regression analyses identified social self-perception as a consistent negative predictor of exhaustion and cynicism, and academic self-perception as a significant positive predictor of academic efficacy. Reflective self-perception mechanisms were found to function as protective factors across all models.

**Conclusions:**

Medical students’ burnout is significantly linked to their perceptions of both academic and social aspects of their competences related to their educational environment. Enhancing self-reflective capacities and fostering a socially supportive learning climate may help reduce burnout risk, particularly among international and female students.

**Supplementary Information:**

The online version contains supplementary material available at 10.1186/s12909-026-08786-8.

## Introduction

Burnout is predominantly a psychological syndrome characterized by emotional exhaustion, cynicism (or depersonalization), and reduced sense of personal accomplishment, resulting from chronic stress and insufficient coping with demanding situations [1]. Several prior studies showed that burnout symptoms are prevalent among medical students, especially among groups in clinical years [2–5]. Based on differences in cultural and institutional context and phase of medical education, the prevalence of burnout among medical students ranges between 10% up to 60% [6, 7]. Thus, burnout of medical students is an educational concern, with complex outcomes, affecting mental well-being of students and deteriorating their academic performance and professional socialization [8]. More particularly, elements of the professional and academic environment, such as workload, quality of teaching and social support appear to be influential on burnout outcomes [9–12]. This may lead to a vicious cycle, since experiences of high academic distress and poor social support trigger burnout symptoms, which increase the likelihood of diminished self-perception leading to increased academic disengagement and distress [13, 14]. Prior findings support this notion showing peer support, encouraging academic environment and helpful university teachers as protective factors [15–17]. Self-perception may be crucial to disrupt this cycle, as it enhances students’ interpersonal competencies, motivation and academic engagement, which may positively affect peer-relations and relations with teachers, thus it serves as protective factor in relation to burnout [13, 14, 18–20].

A widely utilized method to evaluate the educational environment in medical context is the application of a self-report questionnaire, entitled the Dundee Ready Education Environment Measure (DREEM) [21]. Two of its subscales refer to self-reflective mechanisms, one to students’ perceived capabilities to succeed academically and to their ability to develop and maintain supportive social peer relationships [21–24]. As pointed out above, especially social-, and academic self-perception are suggested to buffer the negative effects of psychological and academic distress and reinforce students’ mental well-being and academic engagement [13, 14, 18]. However, the relationship between these self-perceptions and the specific aspect of burnout referred as perceived academic efficacy still needs to be clarified [18–20].

Besides institutional factors, sex, study year and cultural background are also frequently cited correlates of burnout. The prevalence of burnout symptoms is higher for female medical students, particularly for emotional exhaustion, compared to males [25–27]. Students in early years of their education may show signs of burnout, but students in more advanced stages in their education, particularly in the clinical-phase, report more pronounced burnout symptoms [2, 5]. International students appear to be especially vulnerable to burnout, based on their expected adjustment to the host culture [28]. Acculturative stress (e.g., language barrier, homesickness, social integration) is associated with higher prevalence of burnout symptoms and academic struggles [25, 29].

Despite the growing recognition of the importance of institutional and sociodemographic factors, only a few studies have examined the simultaneous contributions of students’ perceptions on institutional quality alongside their self-perceptions to all three main burnout dimensions (exhaustion, cynicism, and perceived academic efficacy) with respect to cultural and sex differences. Former studies showed that overall quality of the learning environment is associated with burnout, but only a few studies differentiated institutional perceptions and reflective self-perceptions and tested their unique contributions to all burnout dimensions [12, 15, 17]. Using the DREEM framework in three multilingual medical programs, we examine whether academic and social self-perceptions uniquely predict the academic efficacy dimension besides perceived teaching, learning, and atmosphere. Furthermore, the perceptions of foreign students regarding their educational environment, together with their academic and social competencies are affected by cultural differences between the original and host culture. Thus, international medical school programs may benefit from a more detailed analysis of the universal factors preventing student burnout. Accordingly, the present study aimed to investigate the relationship between perceived educational environment factors and reflective self-perceptions and burnout outcomes in a multinational medical student sample, controlling for cultural and sex differences. Specifically, we hypothesized that:

1. Poorer perceived quality of teaching and institutional climate will be associated with increased levels of emotional exhaustion and cynicism.
2.  Elevated academic and social self-perceptions were expected to positively predict perceived academic efficacy.
3. Academic and social self-perception will be the strongest predictors of lower-level burnout across all three dimensions.

## Methods

### Participants

Minimum required sample size was calculated based on estimated statistical power for linear regression with a small effect size and a conservative approach (f^2^=0.02, β >=0.95, alpha = 0.05, with 4 predictors in first and 9 in second set) using the Free Statistics Calculators v4.0 online software. A minimum required sample size of 648 was indicated.

To obtain a heterogeneous sample, participants were recruited via the internet by posting invitations on various forums and mailing lists which can only be accessed with university student status. Data was collected during the instruction period (minimum 1 month before starting of the exams) of both the fall and spring semester (September-October and April-May, respectively) of the 2024–2025 academic year. Inclusion criteria were age 18 or above and having currently an active semester in Medical School at University of Pécs. All students participated voluntarily, without receiving any form of compensation. Participants were recruited from all three different language programs of medical school across all 12 semesters of medical education. The research was approved by the relevant ethics committee and was conducted in accordance with the World Medical Association’s Code of Ethics (Declaration of Helsinki). Informed consent was obtained from all participants before starting the online survey.

### Study curricula

Medical education in Hungary offers an integrated six-year medical degree, with a unified curriculum structure for the Hungarian-, English-, and German-language medical programs. The curriculum consists of four different, sequential modules: first two years (semesters 1–4) of basic science module, one year (semesters 5–6) of preclinical studies module, two years (semesters 7–10) of clinical studies module and a final year (semesters 11–12) with clinical clerkships. All three programs are identical in regard of overall duration, progression, and educational objectives, and students are trained within the same institutional infrastructure and clinical environment. The primary distinction between programs lies in the language of instruction and assessment (Hungarian, English, or German), and in the linguistic and cultural background of the student cohorts. In clinical settings, international students receive instruction and are assessed in their program language, while patient communication may involve Hungarian with institutional language support. Thus, there is a highly identical structure and curricular equivalence across the three programs, enabling the comparisons of perceptions on educational environment and burnout dimensions across language groups.

### Questionnaires

The survey included demographic questions (age, sex, study semester) and two validated questionnaires, the Dundee Ready Education Environment Measure (DREEM) and the Maslach Burnout Inventory-Student Survey (MBI-SS). Since both questionnaires were available and validated before in English, German and Hungarian, three versions of the study survey were developed to allow participants to answer questions in their language of education (HUN, EN and GER respectively).

*DREEM* is a widely used questionnaire designed to evaluate students’ perceptions of their educational environment specifically in medical schools and other health professional education settings. It is considered as one of the most suitable self-report measures for this purpose [23, 30, 31]. The instrument is divided into five subscales: students’ perceptions of learning (SPL; i.e. evaluation of their own learning experiences linked to the quality of teaching and the relevance of the curriculum), students’ perceptions of teachers (SPT; i.e. students’ opinions about their teachers in terms of their teaching styles, availability, and support), students’ academic self-perceptions (SAS; i.e. self-assessment of the student’s academic abilities, motivation, and performance), students’ perceptions of the atmosphere (SPA; i.e. perceptions of the institution’s social and academic atmosphere) and students’ social self-perceptions (SSP; i.e. students’ self-evaluations regarding their social skills, peer-relations, and sense of well-being). Each item of this 50-item tool is scored on a 5-point Likert scale (ranging from 0 to 4), with higher scores indicating a more positive educational environment for each subscale.

The 16-item *MBI-SS* was utilized to measure burnout, with three subscales representing different domains of burnout: emotional exhaustion (EE; i.e. the withdrawal of emotional resources due to demanding interpersonal contacts), cynicism (CY; i.e. a negative, callous attitude towards one’s work, colleagues, and patients), and academic efficacy (AE; i.e. students’ sense of competence and effectiveness in their academic abilities) [1]. Responses are provided on a 7-point Likert scale (from 0 to 6) with higher scores for CY and EE and lower scores for AE suggesting burnout. Prior findings showed that item 13 may be ambivalent and unsound and therefore, it was deleted from the MBI-SS [32–34].

### Statistical analysis

The internal consistency for all subscales and all language groups was evaluated using Cronbach’s alpha (see Table 1 and the Supplementary table) and only the DREEM social self-perception subscale in the EN and GER group and DREEM Academic Self-perception in the GER group did not reach the expected value for acceptable reliability. Normality assumptions were tested by Shapiro-Wilk test and non-parametric Kruskal-Wallis tests were performed to test differences between language groups due to violations of normality. That is, a series of independent sample Kruskal-Wallis tests were conducted to compare the three different language groups (HU, EN and GER). Post-hoc pairwise comparisons were made using Bonferroni correction. Based on the differences between the three language groups and lack of normality, partial correlations were conducted (controlling for sociodemographic factors and language) to explore the associations between DREEM and MBI-SS subscales. In particular, to control for the effects of demographic and cultural factors on the relationship between the evaluation of educational environment and level of burnout, the associations between DREEM subscales (SPL, SPT, SAS, SPA, SSP) and MBI-SS subscales (EE, CY and AE) were explored with partial correlation analysis controlling for sex, age, study semester and language group. We used a hierarchical linear regression approach to examine the predictive values of DREEM subscales on burnout dimensions. The same linear regression analyses were performed for all three MBI-SS subscales as the dependent variable in the model. Sex, and language group as variables were dummy coded. First, as step 1, we entered the control variables (sex, age, study semester and language) and in step 2, different aspects of learning environment (DREEM subscales) were added to the model. All regression models were validated by checking multicollinearity and residual assumptions and were found to be acceptable. Most models showed reasonably normal residual distributions, but minor deviations were noted in specific cases of Cynicism and Efficacy. However, based on the sample size, these were not considered as biases for regression coefficients. All data analyses were performed using IBM SPSS statistical software, version 28.0.0.0.

## Results

The final sample comprised 731 medical students (485 females and 10 preferred not to answer) aged between 18 and 42 years (M = 23.12, SD = 3.07). All three language programs were represented [Hungarian (HU), *N* = 143; English (EN), *N* = 280; and German (GER), *N* = 308)] across all semesters (Median = 6; range: 2–13). Table 1 depicts all descriptive statistics regarding the sample and variables of the study.

**Table 1:** Descriptive statistics of the participants and variables included in the study

Variable		Descriptives				
		Ratio	Mean	SD	Skewness	Kurtosis
1	Age (all)		23.12	3.07	0.835	4.853
	Age (HU)		22.60	2.66	1.387	2.980
	Age (EN)		23.65	3.52	0.272	4.078
	Age (GER)		22.87	2.74	1.346	6.998
2	Sex (all)	66.3% female				
	Sex (HU)	69.9% female				
	Sex (EN)	58.2% female				
	Sex (GER)	71.8% female				

			Median	Range (min-max)	Skewness	Kurtosis
3	Study semester (all)		6	2–13	0.304	− 1.047
	Study semester (HU)	basic – 30.8% (*N* = 44) preclinical – 25.9% (*N* = 37) clinical – 31.5% (*N* = 45) final – 11.8% (*N* = 17)	5	2–13	0.529	− 0.630
	Study semester (EN)	basic – 44.6% (*N* = 125) preclinical – 26.8% (*N* = 75) clinical – 25.7% (*N* = 72) final – 2.9% (*N* = 17)	5	2–12	0.676	− 0.434
	Study semester (GER)	basic – 33.4% (*N* = 103) preclinical – 17.9% (*N* = 55) clinical – 44.8% (*N* = 138) final – 3.9% (*N* = 12)	6	2–11	− 0.172	− 1.284

basic = students in the basic module (semesters 1–4); preclinical = students in the preclinical module (semesters 5–6); clinical = students in the clinical studies module (semesters 7–10); final = students in their final year with clinical clerkship (semesters 11–12)

### Language group comparisons

Results showed significant differences across all subscales of both DREEM and MBI-SS, as well as the age and study semester variables (all *p* <.05). No significant difference was found regarding the distribution of sex across groups (*χ²* [2] = 5.072, *p* =.079). Table 2 shows all details and post-hoc pairwise comparisons (with Bonferroni correction). These differences indicate that studying abroad and the cultural background of students influences how they perceive their educational environment and how well they are able to cope with academic challenges.

**Table 2:** Summary of Kruskal-Wallis test results across Language groups

Variable	χ² (2)	*p*-value	Significant Pairwise Comparisons (Bonferroni adjusted *p*)
DREEM Learning	41.976	< 0.001	GER < EN (< 0.001), GER < HU (< 0.001)
DREEM Teachers	35.127	< 0.001	GER < HU (0.005), GER < EN (0.018)
DREEM Academic Self-Perception	12.489	0.002	EN > HU (0.002), EN > GER (< 0.001), HU > GER (< 0.001)
DREEM Atmosphere	85.751	< 0.001	EN > GER (< 0.001), EN > HU (0.001), HU > GER (0.029)
DREEM Social Self-Perception	61.327	< 0.001	GER > EN (0.001), GER > HU (< 0.001)
MBI-SS Exhaustion	20.714	< 0.001	GER > EN (< 0.001), GER > HU (< 0.001)
MBI-SS Cynicism	117.228	< 0.001	GER < EN (0.001), GER < HU (0.008)
MBI- SS Efficacy	15.481	< 0.001	EN > HU (0.001), EN > GER (0.028)
Age	13.815	0.001	GER < EN (< 0.001), GER < HU (< 0.001)
Study Semester	41.164	< 0.001	EN > HU (0.003), EN > GER (< 0.001)
Sex	5.072	0.079	n.s.

All reported p-values are Bonferroni-adjusted where applicable

*HU* students of the Hungarian language program (domestic students), *EN* students in the English language program, *GER *students of the German language program, *n.s.* non-significant

χ² = Kruskal-Wallis test statistic (df = 2)

### Partial correlations

Consistent and statistically significant associations were found between less favourable educational environments and higher levels of burnout (see Table 3). More specifically, all five DREEM subscales were significantly and negatively correlated with Exhaustion (ranging from *r* = −.29 to − 0.35, all *p* <.01), suggesting that students who perceive their educational environment negatively also report feeling emotionally exhausted. Similarly, Cynicism was also significantly and negatively associated with all five DREEM dimensions (ranging from *r* = -.37 to −0.40, all *p* <.01) indicating, that students with poorer perceptions are more likely to have a more negative, callous attitude towards their academic work. The correlations between Efficacy and the DREEM subscales also showed expected directions. Significant positive associations were found (ranging from *r* = -.30 to −0.39, all *p* <.01) indicating that more positive perceptions of the educational environment are associated with elevated feelings of competence and self-efficacy regarding academic work.

**Table 3:** Partial correlations among study variables controlling for age, semester, sex, and Language group

Variable	1	2	3	4	5	6	7	8
1. DREEEM Learning	—							
2. DREEM Teachers	0.744**	—						
3. DREEM Academic Self-Perception	0.652**	0.675**	—					
4. DREEM Atmosphere	0.781**	0.750**	0.750**	—				
5. DREEM Social Self-Perception	0.666**	0.530**	0.668**	0.691**	—			
6. MBI-SS Exhaustion	–0.294**	–0.314**	–0.341**	–0.341**	–0.349**	—		
7. MBI-SS Cynicism	–0.383**	–0.366**	–0.396**	–0.393**	–0.396**	0.579**	—	
8. MBI-SS Efficacy	0.337**	0.299**	0.390**	0.340**	0.333**	–0.167**	–0.234**	—

Values are partial correlation coefficients controlling for age, semester, sex, and language group (dummy-coded)

*p <.05. **p <.001

### Regression analysis

Three hierarchical multiple regressions were conducted to explore educational environment-related predictors of burnout, with Exhaustion, Cynicism, and Efficacy as dependent variables. In each model, as the first step, the control variables were included (age, semester, sex, language group), and in the second step, the DREEM subscales (SPL, SPT, SAS, SPA, SSP) were added as predictors. Multicollinearity diagnostics were in acceptable range (VIF: 1.02–4.32; Tolerance: 0.23–0.98). In Table 4, regression coefficients and model significances of all three regressions were summarized.

**Table 4:** Standardized regression coefficients (β) for predicting MBI-SS subscales Exhaustion, Cynicism, and efficacy

Predictor	Exhaustion β (*p*)	Cynicism β (*p*)	Efficacy β (*p*)
DREEM Learning	0.100 (0.155)	–0.039 (0.540)	0.008 (0.912)
DREEM Teachers	–0.164 (0.002)**	–0.129 (0.009)**	0.044 (0.414)
DREEM Academic Self-Perception	–0.014 (0.810)	–0.026 (0.633)	0.254 (< 0.001)***
DREEM Atmosphere	–0.155 (0.020)*	–0.106 (0.081)	0.042 (0.528)
DREEM Social Self-Perception	–0.223 (< 0.001)***	–0.196 (< 0.001)***	0.111 (0.034)*
Sex (Female)	0.099 (0.004)**	–0.014 (0.658)	–0.117 (< 0.001)***
Study semester	–0.012 (0.768)	0.119 (0.001)**	0.074 (0.070)
Age	–0.017 (0.671)	–0.025 (0.482)	–0.004 (0.927)
Language groups (EN)	–0.072 (0.183)	–0.090 (0.069)	0.005 (0.922)
Language group (GER)	–0.079 (0.150)	–0.336 (< 0.001)***	–0.231 (< 0.001)***
Adj. R²	0.172	0.316	0.180
Model F	15.95***	34.33***	16.87***

Standardized beta coefficients are shown. Dummy variables for language (EN, GER) were entered with HU as the reference

*p* <.05*, *p* <.01**, *p* <.001***

### Exhaustion

Our first model aiming to predict Exhaustion was significant [F(10, 711) = 15.95, *p* <.001], explaining 17.2% of the variance, with significant negative predictors of Teachers, Atmosphere and Social Self-Perception, which had the strongest negative impact. These results indicate that better evaluations of teacher’s work, overall atmosphere and sense of community and belonging were associated with lower levels of emotional depletion.

### Cynicism

Second, Cynicism as dependent variable was analysed and this model was also significant [F(10, 711) = 34.33, *p* <.001], with 31.6% of the variance explained. Two significant negative predictors emerged, Teachers and Social Self-Perception with Study semester as positive predictor, indicating that higher levels of social self-perception and teacher engagement were associated with lower cynicism, while students further along in their studies tended to report higher cynicism.

### Efficacy

The third model, with Efficacy as dependent variable was significant as well [F(10, 711) = 16.87, *p* <.001] and 18% of variance was explained by it. Academic Self-Perception and Social Self-Perception were significant predictors suggesting that academic and social self-reflective mechanisms enhance students’ sense of competence.

## Discussion

The present study investigated how perceived educational environment and academic and social self-perception relate to three main dimensions of burnout among medical students in different language programs. In line with previous research, this study confirmed that students’ burnout is significantly linked to students’ evaluation of their educational and social environment and competences. More specifically, our results showed that all five subscales of the DREEM scale were significantly associated with all three major components of burnout. Negative learning experiences, lower perceived quality of teaching, less favourable institutional atmosphere, decreased academic and social self-perception were associated with higher emotional exhaustion and more negative attitudes towards medical education, while positive perceptions on those dimensions were linked to higher academic efficacy. These findings align with previous findings underlining the importance of a supportive educational environment for students’ psychological well-being and academic engagement [8, 15, 16].

However, most of these previous studies focused on educational environment as one dimension and defined it as a composite of institutional factors. The present study extends this literature by utilizing a multidimensional approach to the educational environment focusing on and comparing different institutional dimensions and self-perceptions (external and internal dimensions). With this approach, we demonstrated, that the self-perception components are uniquely and strongly associated with the academic efficacy dimension of burnout. Moreover, by examining the similarities and differences of three language programs within the same institution, this study provides important insight into how similar curricula may be experienced differently by distinct student populations, thereby highlighting the role of subjective appraisal in academic well-being. Consistently, the strongest effects were found for academic self-perception in relation to academic efficacy, while all other correlations with other environmental dimensions showed rather small effects. This prominent relationship indicates that although there are other influencing factors, students’ own academic self-evaluations play a significant role in shaping their sense of competence and engagement. These findings have also practical implications, as they suggest, that besides structural changes, interventions should target students’ academic confidence and self-perceptions to enhance effectiveness of burnout prevention. In addition to overall associations, we identified the predictive value of specific aspects (i.e. subscales) of the educational environment on different dimensions of burnout to provide more useful information for future burnout intervention and prevention strategies. Self-reflective mechanisms emerged as most important predictors, with Social Self-Perception as the most robust and consistent factor, suggesting that a strong sense of belonging and peer support can be considered as a protective factor against emotional fatigue and disengagement. This is consistent with previous studies indicating that interpersonal connectedness and perceived peer support significantly influence the prevalence of students’ burnout and their mental well-being [13, 14]. Academic Self-Perception was dominant as predictor for students’ self-efficacy, emphasizing the importance of internalized academic competence in the prevention and management of burnout-related decrease of self-confidence and academic engagement [18, 20]. Students’ perceptions about the reputation and attitudes of their teachers, and overall institutional atmosphere were associated with exhaustion and cynicism, showing that educators and educational climate play an essential role in shaping students’ stress-coping and academic adjustment [11, 22, 23].

Comparing international students to domestic students resulted in mixed patterns, for EN and GER groups, where GER students differed significantly from domestic students in all three burnout dimensions, whereas EN students did show more inconsistent distinctions. It is important to note that these differences are driven less by the dissimilarities in curricula and more from language-related cognitive load, cultural adaptation demands, and differences in prior educational socialization, which may influence how students interpret and respond to the same educational environment. These additional challenges faced by international students might impact individuals to different extent resulting in a unique pattern of emotional exhaustion and social self-perception, rather than uniformly affecting all burnout dimensions. These differences between the international student groups may reflect the different expectations towards educational environment based on institutional differences and socialization in their country of origin.

Being a GER student was found to be a negative predictor for both Cynicism and Efficacy suggesting that German medical students are emotionally more engaged and have more positive attitudes towards their education and future profession. On the other hand, they perceive their competences as less adequate and doubt their academic performance. This interesting difference should be a topic for future studies. The mixed results in comparing GER and EN students to domestic students highlight the relevance of cultural background and group heterogeneity to educational experiences and patterns of burnout. GER students mostly share homogeneous educational and cultural background, whereas students in the English program represent a culturally more heterogenous group. This diversity in cultural and educational background also entails greater variability in expectations, learning strategies, and social integration, which in turn may differentially influence perceptions of the educational environment and burnout components. Even if to a limited extent, but these findings still demonstrate, that interventions should consider student population characteristics and incorporate elements to support international students’ adaptation and integration (e.g., mentorship programs, cultural adaptation workshops) to mitigate burnout risk. Results also revealed sex differences, with female students reporting slightly higher Exhaustion and lower academic Efficacy, supporting former findings reporting distinctive stress perception and coping mechanisms for males and females [25, 27]. Elevated emotional depletion and decreased ability to cope with stress alongside with perceived decrease in ability to perform increases burnout and may trigger the vicarious cycle making females more vulnerable to burnout. Accordingly, gender difference-sensitive approaches may be worth to consider in interventions addressing mental health in medical education.

The present study possesses some strengths in using a multi-language sample, which allows a comparative exploration of cultural and institutional variations. Further, utilizing validated and broadly adopted instruments assure the reliability and improve the relevance of the findings.

Nonetheless, some limitations must be acknowledged. First, the cross-sectional study design limits the interpretation of results and restricts causality. Perhaps, future studies collect longitudinal data, which is more suitable for capturing the dynamics of educational experiences and the development and progression of burnout symptoms. Second, the utilization of self-report measures entails the possibility of social desirability and recall bias. Third, while efforts were made to control for confounding factors such as age, sex, and study semester, other influential contextual factors (e.g., academic performance, coping styles) were not assessed. Finally, the reliability of some subscales (e.g., DREEM Social Self-Perception in EN and GER groups) did not reach the expected standard threshold, which affects the strength of certain associations and limits conclusiveness. Fourth, despite the highly similar curriculum across all three language programs, the multilingual education environment might posit an additional unmeasured effect on the observed differences. Classroom dynamics, faculty-students communication or language-related cognitive load and other factors may also contribute to these differences.

Considering the strengths and limitations of this study, future research is needed incorporating longitudinal designs and mixed methods to enable more precise examination of the specific effects and associations between dimensions of academic environment and student burnout. Furthermore, the efficacy of intervention methods strengthening self-reflective capabilities and peer support networks also need to be evaluated, as well as the influence of institutional policies on students’ perception of the learning environment.

## Conclusion

The current study emphasizes the importance of the perceived educational environment and self-reflective mechanisms in shaping medical students’ vulnerability to burnout. Social and Academic Self-Perceptions emerged as protective factors, while negative perceptions of teaching quality and institutional atmosphere contributed to more severe burnout symptoms. These findings highlight the relevance of interventions targeting educational environment and student self-reflection in promoting well-being and preventing burnout in medical education.

## Supplementary Information

Supplementary Material 1.

## Data Availability

The data that support the findings of this study are available from the corresponding author upon reasonable request.
