# Supplementary material for: Associations between the perceived educational environment and burnout symptoms across multilingual medical programs: the role of academic and social self-perception
Source: BMC Med Educ. 2026 Feb 10;26:434. doi: 10.1186/s12909-026-08786-8 (PMC12997865; doi:10.1186/s12909-026-08786-8)
Supplement: Supplementary file 1 — Supplementary Material 1. [file 12909_2026_8786_MOESM1_ESM.docx]

**Supplementary Table**

Descriptive statistics of the variables (subscales) included in the study

|  |  | Descriptives | | | |  | | Reliability | |
| --- | --- | --- | --- | --- | --- | --- | --- | --- | --- |
|  | Variable | Mean | SD | Skewness | Kurtosis | |  | | Cronbach’s alpha |
| 1 | DREEM Learning (all) | 28.10 | 8.01 | - 0.355 | 0.056 | |  | | 0.893 |
|  | DREEM Learning (HU) | 31.71 | 7.46 | - 0.439 | 0.305 | |  | | 0.816 |
|  | DREEM Learning (EN) | 26.16 | 8.52 | - 0.452 | 0.144 | |  | | 0.878 |
|  | DREEM Learning (GER) | 28.18 | 7.17 | - 0.060 | - 0.723 | |  | | 0.794 |
| 2 | DREEM Teachers (all) | 27.37 | 6.81 | - 0.318 | 0.221 | |  | | 0.839 |
|  | DREEM Teachers (HU) | 25.41 | 7.77 | - 0.067 | -0.043 | |  | | 0.851 |
|  | DREEM Teachers (EN) | 26.66 | 6.27 | - 0.156 | 0.691 | |  | | 0.77 |
|  | DREEM Teachers (GER) | 28.91 | 6.47 | - 0.534 | 0.276 | |  | | 0.778 |
| 3 | DREEM Academic Self-perception (all) | 18.34 | 5.84 | - 0.135 | -0.159 | |  | | 8.23 |
|  | DREEM Academic Self-perception (HU) | 17.34 | 5.68 | - 0.104 | - 0.202 | |  | | 0.751 |
|  | DREEM Academic Self-perception (EN) | 17.88 | 6.38 | - 0.071 | - 0.212 | |  | | 0.86 |
|  | DREEM Academic Self-perception (GER) | 19.22 | 5.28 | - 0.113 | - 0.274 | |  | | 0.691 |
| 4 | DREEM Atmosphere (all) | 29.97 | 8.33 | -0.288 | -0.194 | |  | | 0.852 |
|  | DREEM Atmosphere (HU) | 29.89 | 6.86 | 0.097 | - 0.291 | |  | | 0.747 |
|  | DREEM Atmosphere (EN) | 26.56 | 8.48 | - 0.177 | - 0.283 | |  | | 0.861 |
|  | DREEM Atmosphere (GER) | 33.10 | 7.58 | - 0.400 | - 0.150 | |  | | 0.832 |
| 5 | DREEM Social self-perception (all) | 17.17 | 4.67 | -0.308 | -0.097 | |  | | 0.698 |
|  | DREEM Social self-perception (HU) | 17.41 | 4.67 | - 0.142 | - 0.330 | |  | | 0.722 |
|  | DREEM Social self-perception (EN) | 15.54 | 4.59 | - 0.255 | 0.225 | |  | | 0.635 |
|  | DREEM Social self-perception (GER) | 18.53 | 4.29 | -0.428 | - 0.203 | |  | | 0.698 |
| 6 | MBI-SS Exhaustion (all) | 15.35 | 7.33 | 0.007 | -0.814 | |  | | 0.798 |
|  | MBI-SS Exhaustion (HU) | 16.94 | 7.52 | - 0.131 | - 1.099 | |  | | 0.836 |
|  | MBI-SS Exhaustion (EN) | 16.11 | 7.35 | - 0.098 | - 0.679 | |  | | 0.861 |
|  | MBI-SS Exhaustion (GER) | 13.93 | 6.99 | 0.119 | - 0.712 | |  | | 0.833 |
| 7 | MBI-SS Cynicism (all) | 7.24 | 6.71 | 0.777 | - 0.407 | |  | | 0.874 |
|  | MBI-SS Cynicism (HU) | 9.83 | 7.49 | 0.266 | -1.224 | |  | | 0.896 |
|  | MBI-SS Cynicism (EN) | 9.07 | 6.26 | 0.475 | - 0.578 | |  | | 0.836 |
|  | MBI-SS Cynicism (GER) | 4.38 | 5.58 | 1.627 | 2.299 | |  | | 0.863 |
| 8 | MBI-SS Efficacy (all) | 21.39 | 6.72 | - 0.219 | - 0.169 | |  | | 0.833 |
|  | MBI-SS Efficacy (HU) | 22.32 | 4.79 | - 0.211 | - 0.056 | |  | | 0.801 |
|  | MBI-SS Efficacy (EN) | 22.18 | 7.18 | - 0.286 | - 0.108 | |  | | 0.841 |
|  | MBI-SS Efficacy (GER) | 20.23 | 6.88 | - 0.086 | - 0.481 | |  | | 0.833 |
